# The impact of cognateness of word bases and suffixes on morpho-orthographic processing: A masked priming study with intermediate and high-proficiency Portuguese-English bilinguals

**DOI:** 10.1371/journal.pone.0193480

**Published:** 2018-03-12

**Authors:** Montserrat Comesaña, Pauline Bertin, Helena Oliveira, Ana Paula Soares, Juan Andrés Hernández-Cabrera, Séverine Casalis

**Affiliations:** 1 Human Cognition Lab, CIPsi, School of Psychology, University of Minho, Braga, Portugal; 2 Cognitives Sciences and Affectives Sciences Lab, SCALab, Department of Psychology, University of Lille, Lille, France; 3 Department of Psychobiology and Methodology of Behavioral Sciences, University of La Laguna, Tenerife, Spain; Leiden University, NETHERLANDS

## Abstract

Recent studies have suggested that proficient bilinguals show morphological decomposition in the L2, but the question remains as to whether this process is modulated by the cognateness of the morphemic constituents of L2 words and by L2 proficiency. To answer this question was the main goal of the present research. For that purpose, a masked priming lexical decision task was conducted manipulating for the first time the degree of orthographic overlap of the L2 word as a whole, as well as of their morphemic constituents (bases and suffixes). Thirty-four European Portuguese-English bilinguals (16 intermediate and 18 high-proficient) and 16 English native-speaking controls performed the task in English. Results revealed that both groups of bilinguals decomposed words as the native control group. Importantly, results also showed that morphological priming effects were sensitive not only to cross-language similarities of words as a whole, but also to their morphemic constituents (especially, suffixes).

## Introduction

A number of studies in the monolingual domain across languages have shown that visual word recognition is guided by morphological information (e.g., see [1–12]). For instance, an affixed word such as *package* is assumed to be decomposed into its base and suffix components (*pack* + *age*, respectively) at early stages of visual word recognition.

A widely-used task to explore early morphological decomposition effects during lexical access is the masked priming lexical decision task (e.g., [4, 13–19]). In this task, an upper-case target word (e.g., PACK) is preceded by a lower-case prime for 50 ms or less, and participants are asked to decide whether the target is or is not a real word. Primes can be morphologically related to the targets (i.e., derived words, such as *package-PACK*) or unrelated in form and meaning (e.g., *fighter-PACK*). Differences in latencies and accuracy between related and unrelated conditions (priming effects) are taken as evidence of early and automatic morphological decomposition during visual word recognition. Most research using this paradigm has provided evidence for morphological priming effects both when prime-target pairs vary in their degree of semantic transparency (i.e., the extent to which the meaning of a derived word can be deduced from the meaning of their base and suffix; see [20]) and when pairs are not semantically related (i.e., in opaque pairs like corner-CORN), although the magnitude of priming tends to be larger for the former. For this reason, it has been suggested that the effect is morpho-semantic and not simply morpho-orthographic (see, for instance, [5] or [21] for overviews). Importantly, research also showed that priming effects do not occur when the primes end in a set of letters that do not constitute an existing affix in the language, such as in *cashew-CASH* or *turnip-TURN*, in English (e.g., [22]). These results have supported a morphological decomposition account which claims that there is an early segmentation of the word only when the letter string ends in a real affix via an *affix-stripping mechanism* (e.g., [10, 23–28]; see also [29], for a similar approach in the production domain). In the same vein, Diependaele et al. [4] state that there are two distinct mechanisms involved in morphological processing which operate in parallel: a) a purely form-based mechanism, that activates the base whenever the visual input is fully decomposable into a base and a suffix (the morpho-orthographic system); and b) a mechanism that activates the base once the whole word has been activated whenever the visual input is fully decomposable and shares semantic features with its base (the morpho-semantic system). Note, however, that to date there is no consensus among researchers as to the existence of one or two routes in morphological processing (see [30] for more detail).

Controversial issues are even more pronounced in the bilingual domain. For instance, to what extent second language (L2) word recognition is characterized by morphological parsing, and, if so, if it occurs irrespectively of L2 proficiency and cross-language similarities are questions still unanswered in the morphological literature. The present research lies in this domain and aimed to explore how European Portuguese (EP)-English unbalanced bilinguals with different levels of L2 proficiency (intermediate vs. advanced) process L2 morphological derived words (a group of native speakers of English was also included as a control). Interestingly, the cross-language orthographic overlap of morphologically derived words and morphemic constituents (i.e., bases and suffixes) were orthogonally manipulated to better account for the involvement of L1 on L2 morphological processing and, more specifically, to examine the contribution of each morphemic constituent to L2 morphological processing.

Psycholinguistic studies on morphological processing of derived words with bilinguals are scarce (see [31] for a review; and also [32]). A major and hotly debated question in this domain has been whether L2 morphological processing differs from that of L1, and, if so, in what extent (see [33]). Two lines of research can be discerned. The first one suggests that morphological effects upon reading are weaker in the L2 than in the L1. In this vein, several works have shown that adult L2 learners are less sensitive to morphological structure compared to native speakers, and that they rely more on lexical storage than on morphological decomposition. A deeper division between native and non-native languages, irrespective of language idiosyncrasies, is highlighted, and thus the impact of L1 on L2 morphological processing is limited (e.g., [22, 34–37]). For instance, using a masked priming paradigm, Silva [22] showed similar sizes of priming for identity (e.g., bitter-BITTER) and derived conditions (e.g., bitterness-BITTER) in English native speakers, but not in L2 English learners, who revealed reduced priming effects for the derived condition (see also [38, 39], for similar evidence). These findings are consistent with the tenets of Ullman’s model [40], according to which L2 processing depends on the lexical memory system and invokes grammatical computation much less than L1 processing. Conversely, the second line of research suggests that L2 learners decompose words to the same extent that native speakers do (see, for instance, [41–42], and also [43] with verb inflections). Comparing English natives with Dutch and Spanish high-proficient learners of English (L2), Diependaele et al. [41] found significant priming effects, both for the transparent (e.g., viewer-VIEW) and opaque conditions (e.g., corner-CORN), but not for the orthographic control condition (e.g., freeze-FREE, note that–ze in freeze is not a real suffix). Critically, both groups of L2 learners showed the same amplitude in priming effects as native speakers. Therefore, these studies advocate the idea that L1 and L2 morphological processing share the same underlying mechanisms and, thus, differences found across groups (native and non-native) in some studies are explained in terms of L1 influences or cognitive-resource limitations (e.g., [41, 44–50]) rather than lexically-based mechanisms.

However, if we consider that L2 acquisition and processing are dynamic processes (see [50, 51]), it is likely that variables such as L2 proficiency and the degree of cross-language overlap (or cognateness) affect morphological processing, an issue that, up to know, have been little studied despite its importance for the two aforementioned accounts. In fact, there are substantial evidence in the domain of bilingual visual word recognition showing a differential processing of cognates (translation equivalents that share form, e.g., *paper*-*papel*, in English and EP respectively) vs. non-cognates (translation equivalents that only share meaning, e.g., *house*-*casa*; see [52] for an overview), which is modulated by L2 proficiency, as high-proficient L2 learners benefit more from semantic co-activation of cognate words than low/intermediate-proficient L2 learners (see [53, 54]). Differences between cognate and non-cognate words’ processing have been taken as evidence of a non-selective cross-language activation due to an orthographic, phonological, and semantic co-activation (see [55]), as suggested by leading models of visual word recognition (e.g., the Bilingual Interactive Activation Plus model -BIA+, [55 and 56]). According to the BIA+ model, an input word activates competitor words (related in form) in a non-selective or language-independent way. If this is so, and bearing in mind that translation and morphological decomposition are both fast-acting processes who follow a similar time course (see [57–62]), not only the cognateness of whole words would matter, but also that of their morphemic constituents (bases and affixes). It is worth noting that the term cognateness when related to morphemic constituents is employed here to refer exclusively to the degree of form overlap within bases and suffixes.

To our knowledge, very few studies on L2 morphological processing have manipulated the cognate status of whole words [42, 63, 64], and none the cognateness of their constituents (bases and suffixes) despite the fact that not only bases but also suffixes have an essential role in the formation of orthographic representations and lexical access (see for more detail, [65]). One of the studies in which the cognate status of whole-words was manipulated was conducted by Duñabeitia et al. [43]. These authors conducted a masked priming lexical decision study with Spanish-English unbalanced and Basque-Spanish balanced bilinguals. In this study, the degree of overlap of translation equivalents (i.e., cognate vs. non-cognate words), and the language context (primes and targets could belong to the same or different language, e.g., student-STUDY and estudiante[student]-STUDY, respectively) were manipulated. Results revealed a slightly higher (although non-significant) morphological priming effect for cognate words (e.g., student-STUDY [estudiante-ESTUDIAR] over non-cognates (painful-PAIN [doloroso-DOLOR]) in the within language condition (i.e, when primes and targets belonged to the same language), for unbalanced, but not for balanced bilinguals. The difference in the size of priming between cognates and non-cognates was, however, significantly higher when primes and targets belong to different languages (cross-language condition), since the effects here were restricted to cognate words (e.g., estudiante-STUDY > doloroso-PAIN). This result was similar for both groups of participants (unbalanced and balanced). The absence of differences between cognate and non-cognate words in the within-language condition led the authors to claim for a decomposition without translation account. According to this proposal, a fast-acting morpho-orthographic base identification process comes into action, after which the base is mapped onto its corresponding lexical (or lemma-based) representation. Since decomposition tolerates a certain degree of noise in the creation of lexical representations (see the study of McCormick et al. [6] with allomorphs of the base), cognate words would benefit from higher morphological priming effects in the cross-language condition merely due to form overlap. In other words, once decomposition takes place, the isolated base activates other neighbouring lexical representations in a non-selective way (due to the tolerance to slight orthographic changes between the base and its associated lexical representations). Overall, the results replicated those obtained by García-Albea and colleagues [63, 64], a decade and a half earlier with gender number, and verbal inflections with a group of balanced Catalan-Spanish bilinguals. These authors also failed to observe differences in the size of morphological priming effects between cognates and non-cognates in the within-language condition. Besides, in the cross-language condition only cognate words showed morphological priming.

These studies, although interesting, have at least one potential caveat: only the cognateness degree of the bases was taken into account, and nothing was said about the suffixes. In other words, no role has been assigned to suffixes in the morphological cognate effect. Note, however, that our visual word recognition system is efficient at capturing morphological relationships between prime-target word pairs that share suffixes (e.g., darkness-HAPPINESS). The effect appears independently of the degree of segmentation of the prime (e.g., -er-WALKER vs. %%%%er-WALKER vs. baker-WALKER, see [66] for more detail), and of the modality of prime presentation (aurally, see [67]; or visually, [66]). Interestingly, it has also been observed with pseudowords which apparently have a morphological structure like in *quickify* (see [68]). These results seem to provide support for the existence of an affix-stripping mechanism by which suffixes are early stripped off from their bases during visual word recognition, and treated as separated units, as previously mentioned. Thus, even though bases and suffixes have different functional meanings, it is plausible to hypothesize that the latter also benefit from cross-language similarities. This is an issue that we wanted to examine in the present research.

To summarize, in this paper we aimed to explore if non-native speakers of English with different degree of L2 proficiency (intermediate vs. advanced) showed morphological decomposition during the recognition of English derived words and, if so, whether or not morphological processing is modulated by cross-linguistic similarities of morphemic constituents. To do that, the degree of cross-language similarity of bases (B) and suffixes (S) of cognate (C) and non-cognate words (NC) was orthogonally manipulated. Thus, four experimental conditions were created: a) both the base and the suffix were cognates (BCSC, e.g., artista- artist, in EP and English, respectively); b) the base was non-cognate and the suffix cognate (BNCSC, e.g., derrotismo-defeatism); c) the base was cognate and the suffix non-cognate (BCSNC, e.g., simplicidade- simplicity), and d) both the base and the suffix were non-cognates (BNCSNC, e.g., armazenamento-storage). English affixed words worked as primes and were followed by non-affixed English target words (e.g., storage-STORE). In Experiment 1, both groups of EP-English bilinguals carried out a masked priming lexical decision in English. Taking into consideration that morphological decomposition and translation are both fast-acting processes that follow a similar time course in unbalanced bilinguals (see [57–62]), as well as that the cognate effect is modulated by L2 proficiency (see [52–54]), we expected to observe modulations in the size of morphological priming as a function of cross-language similarities of bases and suffixes. Thus, as targets from BCSC and BCSNC conditions are both cognate words that do not differ in the degree of form overlap, e.g., crystal[cristal] and doctor[doutor], respectively), and targets from BNCSC and BNCSNC are both non-cognates that do not differ in the degree of form overlap either e.g., leaf[folha] and milk[leite]), any differences in the size of masked priming would by index of morphological processing due to differences in the degree of form overlap of suffixes of derived primes (crystalline[cristalino], doctorate[doutorado], leafage[folhagem], and milky[leitoso]). (Of note that the degree of form overlap between prime and target was also matched across conditions).

In the Experiment 2, a group of native speakers of English replicated the Experiment 1. Note that, if native speakers of English, who have no knowledge of Portuguese, showed the effect of cognateness (i.e., differences across conditions), this would suggest that other variables rather than cognateness might inadvertently confound the results.

## Ethics statement

The experiments presented here have been carried out in accordance with the code of ethics of the World Medical Association (Declaration of Helsinki) and were approved by the Ethics Committee for Human Research (SECSH 017/2015) of the Research Center on Psychology (CIPsi) at the University of Minho.

## Experiment 1

### Method

#### Participants

Thirty-four European Portuguese (L1)-English (L2) bilinguals (31 females, *M*_age_ *=* 25; *SD =* 6.7) took part in the experiment. Participants were recruited at the Wall Street English Institute of Oporto (58.8%) and at the University of Minho, and had normal or corrected-to-normal vision. All participants signed a consent form for the research and filled the Portuguese translation of background tests published by Casalis, Commissaire, and Duncan [2]. These tests evaluate participants’ L2 proficiency and allowed us to constitute the two groups of high and intermediate English bilinguals in this study. In a lexical test, participants had to translate 150 Portuguese words into English and in a spelling test they had to choose the correct spelling between two possible candidates. Both groups comprise 18 participants each, although two participants from the intermediate group were not included in the final sample because of a high percentage of errors made during the experiment (more than 50%). Therefore, in the final sample, the intermediate proficient group was constituted by 16 participants (*M*_proficiency_ = 72.25; *SD* = 5.79) and the high proficient group by 18 participants (*M*_proficiency_ = 87.57; *SD* = 2.68; differences were significant; *p* < .001). Of note, the assignment of participants from the Wall Street Institute to the two groups of proficiency according to the results in our L2 proficiency tests matched that provided by the teachers. Participants also filled the Language History Questionnaire (LHQ, [69]). The average age of acquisition of English as a second language was 9.21 years *(SD =* 1.86). The LHQ also allow to collect subjective ratings of reading, writing, speaking, and listening skills, based on a 7-point Likert scale (from 1 = *very poor* to 7 = *native-like*). Subjective ratings mean was 5.1 (*SD* = 0.57) and 5.54 (*SD* = 0.54) in the intermediate and high-level groups, respectively (*p* < .05). In order to ensure that participants knew the stimuli, a recognition task composed of the 56 experimental target words was proposed at the end of the experimental task. The percentage of words participants reported to be unfamiliar with was 5.6% (*SD* = 3.66) in the intermediate level group and 2.5% (*SD* = 2.27) in the high-level group. All participants were volunteers and none were paid to take part in the experiment.

#### Materials

Fifty-six English-European Portuguese (EP) translation equivalent words were selected. These words were assigned to four experimental conditions according to the degree of orthographic overlap of bases and suffixes across languages: Cognate Base and Cognate Suffix (BCSC, e.g., passage-*passagem*, in English and EP, respectively); Non-cognate Base and Cognate Suffix (BNCSC, brokerage-*corretagem*); Cognate Base and Non-cognate Suffix (BCSNC, e.g., rarely-*raramente*); and Non-cognate Base and Non-cognate Suffix (BNCSNC, e.g., blindly-*cegamente*). The cognateness degree of bases and suffixes were calculated using the NIM database [70]. NIM computes the Normalized Levenshtein Distance (NLD), which ranges from 0 (not similar at all) to 1 (exactly the same). As expected, there were no differences in the mean of orthographic overlap of word bases between conditions BNCSC and BNCSNC (.13 and .18, respectively, *p* = .46) or between conditions BCSNC and BCSC (.79 and .88, respectively, *p* = .15). Likewise, there were no differences in the suffixes between conditions BNCSC and BCSC (.55 and .64, *p* = .31) or in the suffixes between conditions BNCSNC and BCSNC (.06 and .12, *p* = .19). Differences were only significant across conditions when they varied either in the type of cognate base or in the type of cognate suffix (all *p*s < .001), as expected. For instance, the BCSC and BCSNC conditions vary in the suffix type and, thus, differences when comparing the cognateness degree of the suffixes were significant. In addition, when counting the number of times a given base appeared in the CELEX corpus (as in the N-Watch database, [71]), no differences were found across conditions (*p* = .22). The same applies to suffixes (*p* = .17). Experimental conditions were also matched in base frequency (*p* = .39) and morphological family size (*p* = .54).

Fifty-six English derived words were used as primes and matched across conditions for logarithmic frequency per million words, length in number of letters, summed log bigram (SLBF), and number of orthographic neighbours (all *p*s > .20; differences as a function of phonological neighbours were marginal [*p* = .077]; see Table 1). These values were taken from the N-Watch database [71].

**Table 1:** Mean and standard deviation (in parentheses) of logarithmic frequency (LogF), length, summed log bigram frequency (SLBF), number of orthographic (N) and phonological (PN) neighbours of prime and target words in each experimental condition. The mean and standard deviation of Base frequency (Base F) and Family size (F. Size) were also included for targets.

	Prime					Target						
	LogF	Length	SLBF	N	PN	LogF	Length	SLBF	N	PN	Base F.	F. size
**BCSC**	0.92 (0.62)	8.29 (1.86)	19.06 (6.18)	0.57 (0.94)	1.33 (1.23)	1.08 (0.75)	5.93 (1.77)	13.01 (3.91)	3.71 (5.28)	8.77 (9.35)	3.21 (3.81)	3.85 (3.80)
**BNCSC**	0.55 (0.55)	7.43 (1.55)	16.80 (2.47)	1.36 (1.69)	3.64 (3.61)	1.34 (0.78)	4.86 (1.23)	11.02 (3.1)	5.86 (4.9)	13.7 (10.3)	4.86 (4.67)	6.00 (4.99)
**BCSNC**	0.90 (0.61)	8.5 (1.83)	19.93 (6.45)	0.69 (1.18)	1.58 (2.19)	1.42 (0.57)	5.43 (0.94)	11.83 (2.57)	2.71 (3.73)	6.08 (5.09)	3.64 (1.78)	4.57 (1.80)
**BNCSNC**	0.59 (0.54)	8.43 (1.83)	19.75 (6.83)	0.50 (0.94)	1.30 (1.89)	1.26 (0.63)	5.36 (1.28)	11.94 (3.24)	3.64 (3.71)	9.23 (5.9)	3.29 (2.23)	3.92 (3.46)

Note: BCSC = Base Cognate Suffix Cognate; BNCSC = Base Non-cognate Suffix Cognate; BCSNC = Base Cognate Suffix Non-cognate; BNCSNC = Base Non-cognate Suffix Non-cognate

As targets, we used the English bases of the English derived words (e.g., brokerage-BROKER). Most of them were nouns that also function as verbs (61%). The mean cognateness degree of target words considered as a whole did not differ between conditions BCSNC and BCSC (cognate targets; .71 and .75 respectively, *p* = .60) or BNCSNC and BNCSC (non-cognate targets; .17 and .17 respectively, *p* = .97), as expected. Thus, any difference between BCSNC and BCSC targets or between BNCSNC and BNCSC targets would be an index of morphological processing since they only vary in the suffix type of related primes. Like the primes, targets were matched across conditions in logarithmic frequency per million words, length, bigram frequency, orthographic neighbors, and phonological neighbors (all *p*s > .12; see Table 1). They were also matched in word class (*p* = .79). When considering the Portuguese translations of the English targets (e.g., *corretor* [BROKER]), no differences were found across conditions in logarithmic frequency or length (all *p*s > .87). These values were taken from the Procura-PALAvras [P-PAL] database [72]. English targets were preceded by morphologically related primes (the English derived words; e.g., brokerage-BROKER) or by unrelated primes (no relation in form or meaning; e.g., attendant-BROKER). Most of them were nouns that also function as other word classes (71.4%). Importantly, prime-target orthographic overlap was similar across conditions both when they were related (*p* = .60) and unrelated (*p* = .57). In addition, there were no differences across related conditions when it comes to prime-target phonological overlap (*p* = .19). Related and unrelated prime words were matched in log frequency and length both in English (all *p*s > .13) and Portuguese (all *p*s > .15). The entire set of materials is presented in Appendix A. Due to the nature of the lexical decision task, 56 pseudowords were created from words selected from the same population as the experimental targets using the Wuggy software [73]. For instance, the word *beat* yielded the pseudoword *leat*. This pseudoword was either preceded by a word derived from the word used to create the pseudoword (e.g., *beater*-LEAT) or by an unrelated word (e.g., *intruder-*LEAT). Two lists were created to counterbalance prime-target pairs. The order of stimulus presentation from each list was randomized for each participant.

#### Procedure

Participants were individually tested in a silent room to perform the lexical decision task (LDT) in English as well as the LHQ, spelling, translation, and familiarity tasks mentioned in the participants’ section. In the LDT, participants were asked to decide as quickly and accurately as possible whether a given string of letters was a real English word using two different response keys (*M* and *Z* corresponding to *Yes* and *No* answers). Stimulus presentation and data recording were controlled by the DMDX software [74].

For each trial, a mask presented at the centre of the screen for 500 ms (a series of #) was followed by a lowercase prime presented in 12-pt Courier New for 50 ms (3 ticks in the 60-Hz CRT monitor). The prime was then replaced by an uppercase target word, which remained on screen until participants responded or 2500 ms had elapsed. The inter-trial interval was 1,133 ms (68 ticks). There were two blocks, a practice block with 10 practice trials (5 words + 5 pseudowords) and an experimental block with 112 experimental trials (56 words + 56 pseudowords). The task started with the practice block and was followed by the experimental block. One break was included after 56 trials had been presented. The entire procedure took approximately 35 minutes.

### Results and discussion

Errors and lexical decision times below 250 ms or above 2000 ms were excluded from the latency analyses as well as data for the items that participants reported to be unfamiliar with. Reaction Times (RTs) more than 2 standard deviations (SDs) above or below participants’ means per condition were also removed. As a result, 5.3% of the data were eliminated. Besides, participants with more than 25% errors were excluded (three from the List 1 and four from the List 2). The mean lexical decision times and errors percentage per condition are displayed in Table 2.

**Table 2:** Mean lexical decision times (RTs; in milliseconds) and proportion of errors (E) per condition (standard deviations between parentheses) in bilinguals with high and intermediate levels of L2 proficiency.

	BCSC		BNCSC		BCSNC		BNCSNC	
	Rel.	Unr.	Rel.	Unr.	Rel.	Unr.	Rel.	Unr.
**High**	611 (124.4)	685 (131.6)	616 (126.3)	679 (135.2)	592 (99.6)	608 (99.3)	658 (137.1)	711 (136.2)
	0.03 (0.18)	0.06 (0.23)	0.06 (0.23)	0.12 (0.33)	0.01 (0.09)	0.02 (0.15)	0.06 (0.24)	0.12 (0.33)
**Intermediate**	678 (141.3)	748 (155.1)	651 (119.5)	729 (152.0)	632 (87.8)	675 (96.0)	743 (158.7)	759 (166.1)
	0.04 (0.19)	0.02 (0.13)	0.13 (0.33)	0.12 (0.32)	0.04 (0.19)	0.03 (0.16)	0.13 (0.34)	0.12 (0.32)

Note: Rel. stands for Related prime-target conditions and Unr. stands for Unrelated prime-target conditions.

We analysed RTs and accuracy for word targets with linear mixed effects (lme) models. The lme on RTs were conducted with participants and items as crossed random factors, and with random intercept and all repeated measure factors with random slope per subject and not per item (see [75]). For accuracy, we used a generalised lme with logistic link function and binomial variance. The models were fit using the lme4 R library ([76]) and LmerTest library in order to contrast simple effects with differences of least squares means. There was no averaging of the data prior to the analyses.

We first investigated the presence of a significant 2×2×2×2 interaction between the design factors Proficiency level (high|intermediate), Cognate Base Status (BC|BNC), Cognate Suffix Status (SC|SNC), and Prime type (morphologically related|unrelated). The four-way interaction was significant for the RT data, *F*(1, 1554) = 3.94 *p* < .05 (degrees of freedom with Satterthwaite approximation), but not for the accuracy data. We investigated the interaction in the RTs by simple effects estimation with differences of least squares means as Duñabeitia et al. (2013). The results are summarised in Table 3. The first eight lines present the size of priming effects per experimental condition in each group of L2 proficiency (High and Intermediate) and their significance. In the following lines, comparisons of size priming effects between groups per experimental condition and between experimental conditions are presented along with their significance.

**Table 3:** Individual priming effects [RT] and comparisons in Experiment 1.

*Effect 1*	*Effect 2*	*RT (ms)*	*t*	*p*_*BH*_*-value*
**BC | SC | High**		**77**	**6.54**	**<0.0001**
**BC | SC | Intermediate**		**60**	**3.87**	**0.0004**
**BNC | SC | High**		**64**	**5.35**	**<0.0001**
**BNC | SC | Intermediate**		**76**	**4.70**	**<0.0001**
**BC | SNC | High**		19	1.93	0.0965
**BC | SNC | Intermediate**		**35**	**2.66**	**0.0194**
**BNC | SNC | High**		**62**	**4.56**	**<0.0001**
**BNC | SNC | Intermediate**		13	0.71	0.5613
**BC | SC | High**	BC | SC | Intermediate	17	1.57	0.1947
**BNC | SC | High**	BNC | SC | Intermediate	-12	-0.02	0.9852
**BC | SNC | High**	BC | SNC | Intermediate	-16	-0.65	0.5703
**BNC | SNC | High**	BNC | SNC | Intermediate	**49**	**2.53**	**0.0256**
**BC | SC | High**	BC | SNC | High	**58**	**3.34**	**0.0028**
**BNC | SC | High**	BNC | SNC | High	2	0.48	0.6628
**BC | SC | Intermediate**	BC | SNC | Intermediate	25	0.91	0.5162
**BNC | SC | Intermediate**	BNC | SNC | Intermediate	**53**	**2.85**	**0.0124**
**BC | SC | High**	BNC | SC | High	13	0.80	0.5511
**BC | SC | Intermediate**	BNC | SC | Intermediate	-16	-0.77	0.5511
**BC | SNC | High**	BNC | SNC | High	-43	-2.00	0.0920
**BC | SNC | Intermediate**	BNC | SNC | Intermediate	22	1.30	0.2998
**BC | SC | High**	BNC | SNC | High	15	1.27	0.2865
**BC | SC | Intermediate**	BNC | SNC | Intermediate	47	2.16	0.0667
**BC | SNC | High**	**BNC | SC | High**	**-45**	**-2.52**	**0.0284**
**BC | SNC | Intermediate**	BNC | SC | Intermediate	-41	-1.66	0.1651

*Notes*: BCSC (Base Cognate and Suffix Cognate); BNCSC (Base Non-cognate and Suffix Cognate); BCSNC (Base Cognate and Suffix Non-Cognate); BNCSNC (Base Non-Cognate and Suffix Non-Cognate); High (High-proficient bilinguals); Intermediate (Intermediate proficient bilinguals). Significant effects are presented in bold.

The interaction reflected priming effects for those conditions whose suffixes are cognates (BCSC and BNCSC, all *p*s < .001), regardless of the level of L2 proficiency (note that no differences in the size of priming effects were observed between these conditions in both proficiency groups, *ps* = .55). Interestingly, the size of priming effects for these conditions were twice as large as those observed for conditions with non-cognate suffixes (BCSNC and BNCSNC, see Table 3). Indeed, the mean of the priming effect size for conditions with cognate suffixes was 69 ms whereas that for non-cognate suffixes was 32 ms, considering both groups of bilinguals. This result was also supported by the two-fold interaction between cognate suffix status and prime type, *F*(1, 1545.20) = 13.29; *p* = .000.

Conversely, when the suffixes are non-cognates (BCSNC and BNCSNC), priming effects seem to be modulated by L2 proficiency, as only high-proficient bilinguals showed morphological priming in the BNCSNC condition (*p* < .001). The size of priming in the BCSNC condition approached significance (*p* = .09) in this group, although it did not differentiate from that observed with the intermediate proficient bilinguals (*p* = .57). Moreover, it is also important to stress here that while in the high-proficient group the interaction showed differences in the size of morphological priming between conditions whose bases were cognates (BCSC and BCSNC, *p* = .002), in the intermediate proficient group, the differences were only observed between conditions whose bases were non-cognates (BNCSC and BNCSNC, *p* = .01). Note that differences within conditions that share a cognate base or a non-cognate base can be taken as an index of suffix morphological processing, since targets from these conditions did not differ either in the degree of orthographic overlap across equivalent translations, or in the degree of orthographic and phonological overlap between prime (derivate words) and targets. Finally, although differences in the size of priming effect between conditions that vary in the type of base (BCSC vs. BNCSC and BCSNC vs. BNCSNC) were not significant, a two-fold interaction between cognate base status and cognate base suffix, *F*(1, 49.66) = 11.22; *p* = .001), showed faster responses for words with cognate bases than for words with non-cognate bases when the suffixes were non-cognates (*p* < .001).

For the accuracy data, the results revealed a main effect of Cognate Base Status, χ^2^(1) = 10.008; *p* = .001, showing that participants committed fewer errors to words with a cognate base than to words with a non-cognate base (i.e., a cognate facilitation effect for cognate words). Besides, the two-fold interaction between Proficiency and Prime type reached statistical significance, χ^2^(1) = 6.83; *p* = .008, showing that morphological priming was restricted to high-proficient bilinguals, *t*(1, 1820) = -2.71; *p* = .04.

Overall, these findings were consistent with previous studies showing that L2 learners exhibit an early morphological decomposition when processing L2 words (e.g., [40, 41]). However, and contrary to what Duñabeitia et al. [42] had observed in the within-language condition (i.e., student-STUDY), in our data the whole-word cognateness as well as the cognateness of words’ morphemic constituents (especially their suffixes) seem to play a crucial role in the recognition of L2 words. In fact, when it comes to latency data, priming effects were observed both when the entire word was a cognate (i.e., when both bases and suffixes were cognates, the BCSC condition), and a non-cognate (i.e., when both bases and suffixes were non-cognates, the BNCSNC condition), although only in the high-proficient group (both *p*s < .001). Morphological priming effect in the intermediate proficient group was, however, restricted to the BCSC condition (*p* < .000). Interestingly, there were no differences in the size of priming effects between conditions with cognate suffixes in both groups (BCSC and BNCSC, both *p*s = .55). Furthermore, conditions with cognate suffixes revealed larger masked priming effects than conditions with non-cognate suffixes (BCSNC and BNCSNC). Thus, it seems to be not strictly necessary to use primes and targets from different languages to observe morphological cross-language effects during the recognition of L2 words. Moreover, the findings clearly suggested that the cognateness degree of morphemic constituents seems to be an appropriate strategy to explore the cognitive mechanisms that underlie non-native morphological processing. These results (although not entirely as expected) led us to develop a tentative theoretical account (the Co-activation of Segmentation and Translation account [COST], see S1 Fig), by which two early and fast-acting processes (translation and decomposition) come into operation when processing L2 words which bases and/or suffixes were either cognates or non-cognates, to account for the morphological priming effects observed. We will come back to this account later on.

Another result that was at odds with the results found by Duñabeitia et al. [42] has to do with the differences observed between intermediate and high-proficient bilinguals. In fact, only the latter group showed morphological priming effects for cognate and non-cognate words both in RTs and error data. This finding may be indicating a refinement in L2 morphological decomposition process as L2 proficiency increases. However, before establishing firm conclusions, it is important to rule out the possible influences of artefacts in the materials. Experiment 2 was designed to explore this issue. A control group of native speakers of English with no knowledge of European Portuguese or other languages (especially Romance languages) replicated Experiment 1. Thus, if the results cannot be ascribed to artefacts in the materials, then no differences should be observed as a function of cognate base and cognate suffix status. Note that words from the four experimental conditions (BCSC, BCSNC, BNCSC, and BNCSNC) only differ in the degree of cognateness (considering whole words or their bases and suffixes).

## Experiment 2

### Method

#### Participants

Sixteen undergraduate students of Psychology (10 women and 6 men) from the University of Kingston (London, United Kingdom) took part in the experiment (*M*_age_ = 22, *SD* = 2.8). They were native speakers of English and had normal or corrected-to-normal vision. None had any knowledge of European Portuguese or other languages. All participants signed a consent form for the research and received eight pounds for their participation.

#### Materials and procedure

The material set and the procedure were the same as in Experiment 1.

### Results and discussion

Data corresponding to incorrect responses were discarded from the analysis (7.9%). Furthermore, reaction times more than two standard deviations above or below the participants’ mean in each condition were removed (1.94%). Mean lexical decision times and errors percentages per condition are displayed in Table 4.

**Table 4:** Mean lexical decision times (RTs; in milliseconds) and proportion of errors per condition (standard deviations between parentheses) in native speakers of English.

	BCSC		BNCSC		BCSNC		BNCSNC	
	Rel.	Unr.	Rel.	Unr.	Rel.	Unr.	Rel.	Unr.
**RTs**	668 (207.9)	699 (187.0)	632 (179.7)	721 (235.4)	651 (226.2)	674 (178.8)	672 (208.1)	704 (208.5)
**Errors**	0.04 (0.21)	0.13 (0.33)	0.07 (0.26)	0.10 (0.29)	0.02 (0.13)	0.04 (0.19)	0.06 (0.24)	0.12 (0.32)

Note: Rel. stands for Related prime-target conditions and Unr. stands for Unrelated prime-target conditions.

We investigated the presence of a significant three-fold interaction between Cognate Base Status (BC|BNC), Cognate Suffix Status (SC|SNC), and Prime type (morphologically related|unrelated) factors. As expected, the interaction was not significant, *F*(1, 732.44) = 0.62, *p* = .43 (degrees of freedom with Satterthwaite approximation). Thus, the results observed previously with bilinguals were not driven by the existence of uncontrolled variables in the sets of items (cognateness of bases and suffixes). The main effect of Prime type was, however, significant, *F*(1, 731.98) = 12.83 *p* < .001, as expected. This effect showed that words preceded by morphologically related primes were read faster than those preceded by unrelated primes.

The analyses on the error data were consistent with what was observed on the latency data, as there was only a significant main effect of Prime type χ^2^(1) = 6.83; *p* = .008. This effect revealed that participants made fewer errors with targets preceded by morphologically related primes than by unrelated primes (see Table 4).

## General discussion

The main objective of the present study was to pin down the factors that influence early segmentation during L2 word recognition. Specifically, we wanted to explore whether L2 morphological processing is affected by the degree of cognateness of whole words and their morphemic constituents, as well as by L2 proficiency. To that aim, intermediate and high-proficient EP-English bilinguals performed a masked priming lexical decision task in which English target words could be preceded by English related (morphologically derived) or unrelated primes. Morphologically derived primes varied in the cognateness degree of their morphemic constituents (i.e., they could belong to the BCSC, BNCSC, BCSNC, or BNCSNC conditions). A group of English native speakers (the control group) performed the same task in order to rule out the existence of artefacts in the materials. The findings were clear-cut: masked morphological priming effects were found in bilingual and monolingual groups. This calls into question the idea that L2-derived words are decomposed and processed differently from those in the native language (e.g., [34, 39, 40]). Interestingly, in bilinguals, morphological priming effects were modulated by the cognateness of morphemic constituents, especially that of suffixes (words with cognate suffixes produced larger priming effects than words with non-cognate suffixes). Besides, when the suffixes were non-cognates, cognate words were faster and better recognized than non-cognate words (BCSNC < BNCSNC, the typical cognate facilitation effect). These results challenge the view that there is a decomposition process without translation that is insensitive to the degree of form overlap between derived primes and their translations [see 42].

Overall, the results sustain the hypothesis of the existence of similar underlying mechanisms that guide non-native and native morphological processing (differences in non-native compared to native morphological processing are due to influences of one language over the other, as inferred from the effect of cognateness on the recognition of L2 words), and that translation and segmentation processes co-occur in a fast and automatic way, at least when target words are in the L2. Note that cognate effects are usually greater in a forward (L1-L2) than in a backward direction (L2-L1) (see [77–80]) and, thus, it would be possible to observe a differential pattern of results if L2 learners had performed the task in their L1, an issue that we wanted to explore in ongoing works.

An important aspect in our findings is that they bring into question what masked priming is telling us about the structure of the bilingual lexicon. In fact, the existence of a fast-acting and automatic decomposition process, that is sensitive to the cognate degree of morphemic constituents, provides further evidence to the idea of an integrated lexicon characterized by a non-selective language activation (as the BIA+ model of visual word recognition holds, see [56] and [55]). Thus, it seems that, when a given word such as *preacher*[pregador] is encountered, it is segmented into its base + suffix pair (preach + -er), and stored form-based representations for these elements are activated. At the same time, the whole word seems to be translated into the other language and the resulting translation is also segmented into its morphemic constituents (prega + -dor). Connections between stored form-based representations that share their form are activated, facilitating their access (see the COST account, S1 Fig).

If we are right, morphological priming effects would be driven by the interaction of decomposition and translation processes and, thus, the properties of lexical representations may affect processing. However, under this view it would be expected that the greater the degree of orthographic overlap the higher the size of morphological priming, a result that we failed to observe in latency data (in the analysis on errors, we observed a cognate facilitation effect by which cognate words were recognized more precisely than non-cognate words). In fact, the strongest priming effects were to words with cognate suffixes. Therefore, why did suffix cognateness have a higher impact on the size of masked priming than base cognateness, or even whole-word cognateness? Note that the preferential processing observed for words whose suffixes were cognates was the same for both high- and intermediate-proficient bilingual groups. Although this result was admittedly surprising, one possibility is to assume that during reading suffixes are the fastest identified constituents as higher-level functional units by some kind of affix-segmentation process (see [25]) to enable the isolation of bases. This affix-segmentation process may be sensitive to cross-language form overlap of sublexical units, and thus the activation that suffixes receive from whole words would be higher when these words are cognates (because, instead of one, two lexical representations are activated via translation). The same would be true for bases, but because they are lexical entities they might compete with each other and the level of activation would thus be reduced. Notwithstanding, we recognize that further research is needed to corroborate this hypothesis, for instance manipulating the SOA and the semantic relatedness of cognate bases. In any case, the present research is not the only one sustaining the COST account. In an interesting study developed by Zhang et al. [59], Chinese (L1)-English (L2) bilinguals were asked to perform a masked priming lexical decision task in English. Primes and targets were unrelated (e.g., east-thing) but, critically, their Chinese translations could be morphologically related (e.g.,东西–东). The authors hypothesized that, if translation occurred in a fast and automatic way, then masked priming effects would be observed for the related pairs in the native language due to the existence of what they called a *hidden morphological repetition effect*, as it was the case (see also [62, 63] for converging electrophysiological evidence).

The findings obtained in Zhang et al.´s study [59] and, particularly, in the present research, rule out the other possible theoretical account proposed by Duñabeitia et al. [42] (decomposition without translation account). According to it, no differences would be expected as a function of cognate words since translation would occur at an abstract level of representation (note that the cognate effect is mainly driven by form overlap between translation equivalents and thus there is no reason to expect an advantage if they are not translated before or simultaneously to segmentation).

However, why do our results with derived words contrast with those observed in the Duñabeitia et al.’s [42] study? These authors asked unbalanced Spanish-English bilinguals to perform a masked priming lexical decision task in English (Experiment 1). Cognate and non-cognate targets (e.g., STUDY[estudiar] vs. PAIN[dolor], in English and Spanish, respectively) were preceded by morphologically related (student vs painful) or unrelated primes. The procedure in this first experiment was similar to the one employed in the present research, with the exception of the fact that the authors did not consider the degree of overlap of words’ constituent morphemes (only that of whole words). The authors failed to show modulations in priming effects as a function of cognateness, although a numerical advantage of 8 ms was observed for cognate words. A relevant aspect of Duñabeitia et al.’s study is that participants’ L2 proficiency was slightly lower than that of our participants from the intermediate L2 proficiency group (4.91 vs. 5.1, respectively), precisely the participants who failed to show cognate effects in the error data. This fact could partially explain the apparent inconsistency across studies. Note that modulations in our research were mainly explained by the cognateness of suffixes (an issue that Duñabeitia et al. did not take into account). Indeed, a closer inspection of the materials used by the authors revealed that both cognate and non-cognate word groups have words with cognate suffixes. This might help to explain the absence of differences between cognate and non-cognate words (note that we also failed to observe differences in the size of masked priming effects between cognate and non-cognate whole words when they were characterized by cognate suffixes—BCSC and BNCSC). In any case, the present study is the first showing modulations in the morphological priming effects as a function of L2 proficiency, as only high-proficient bilinguals showed priming effects for cognate and non-cognate words both in the latency and error data. These results suggest that as the level of L2 proficiency increases the morphological processing becomes more refined.

In sum, the present results support the view that bilinguals decompose words as native speakers do, and that morphological priming effects in a non-native language are sensitive to the cognateness of morphemic constituents, thus giving support to the COST account. Hence, and contrary to what Duñabeitia et al. [42] have suggested, there is an important involvement of the translation process in masked morphological priming, regardless of whether languages share or not the same script (see Zhang et al., [60], for consistent results with Chinese-English bilinguals). A critical issue for future bilingual studies would be to clarify why masked priming effects are mainly modulated by the degree of formal overlap of suffixes across languages, as well as to assess whether similar patterns of results emerge in the two directions of translation.

## Supporting information

S1 Appendix (DOCX)

S1 Dataset (XLSX)

S1 Fig Graphical illustration of the co-occurrence of segmentation and translation account. Inputs per word-type are in English (L2): BCSC (preacher, meaning *pregador* in EP [L1]); BNCSC (player, meaning *jogador*); BCSNC (dancer, meaning *dançarino*); and BNCSNC (header, meaning *cabeçalho*). B stands for Base, S stands for Suffix, C stands for Cognate and NC stands for Non-cognate. Arrows represent facilitatory connections. Stronger links are represented by thick arrows.
(PDF)
