# Supplementary material for: The impact of cognateness of word bases and suffixes on morpho-orthographic processing: A masked priming study with intermediate and high-proficiency Portuguese-English bilinguals
Source: PLoS One. 2018 Mar 12;13(3):e0193480. doi: 10.1371/journal.pone.0193480 (PMC5846768; doi:10.1371/journal.pone.0193480)
Supplement: S1 Appendix — (DOCX) [file pone.0193480.s001.docx]

**Appendix**

Stimuli used in the experiments (European Portuguese translations are within brackets)

| **Condition** | **Target** | **Related Prime** | **Unrelated Prime** |
| --- | --- | --- | --- |
| **BCSC** | **Repulse (repulsa)** | Repulsive (repulsivo) | Poetic (poético) |
| **BCSC** | **Navigate (navegar)** | Navigator (navegador) | Anchorage (ancoragem) |
| **BCSC** | **Penitent (penitente)** | Penitential (penitencial) | Treatment (tratamento) |
| **BCSC** | **Pass (passar)** | Passage (passagem) | Dentist (dentista) |
| **BCSC** | **Explode (explodir)** | Explosive (explosivo) | Vestment (vestimenta) |
| **BCSC** | **Diverge (divergir)** | Divergent (divergente) | Productive (produtivo) |
| **BCSC** | **Preach (pregar)** | Preacher (pregador) | Defensive (defensivo) |
| **BCSC** | **Race (raça)** | Racism (racismo) | Pianist (pianista) |
| **BCSC** | **Crystal (cristal)** | Crystalline (cristalino) | Digestive (digestivo) |
| **BCSC** | **Gene (gene)** | Genetic (genético) | Specialist (espcialista) |
| **BCSC** | **Assist (assistir)** | Assistant (assistente) | Distinctive (distintivo) |
| **BCSC** | **Function (função)** | Functional (funcional) | Aromatic (aromático) |
| **BCSC** | **Base (base)** | Basic (básico) | Animator (animador) |
| **BCSC** | **Art (arte)** | Artist (artista) | Modifier (modificador) |
| **BNCSC** | **Play (jogar)** | Player (jogador) | Drainage (drenagem) |
| **BNCSC** | **Fake (falsificar)** | Faker (falsificador) | Payment (pagamento) |
| **BNCSC** | **Defeat (derrota)** | Defeatism (derrotismo) | Collector (cobrador) |
| **BNCSC** | **Ravish (arrebatar)** | Ravishment (arrebatamento) | Forwarder (expedidor) |
| **BNCSC** | **Sell (vender)** | Seller (vendedor) | Claimant (requerente) |
| **BNCSC** | **Jump (saltar)** | Jumper (saltador) | Slippage (derrapagem) |
| **BNCSC** | **Pack (embalar)** | Package (embalagem) | Fighter (lutador) |
| **BNCSC** | **Appraise (avaliar)** | Appraiser (avaliador) | Builder (construtor) |
| **BNCSC** | **Read (ler)** | Reader (leitor) | Coinage (cunhagem) |
| **BNCSC** | **Dream (sonhar)** | Dreamer (sonhador) | Footage (metragem) |
| **BNCSC** | **Leaf (folha)** | Leafage (folhagem) | Judgment (discernimento) |
| **BNCSC** | **Stop (parar)** | Stoppage (paragem) | Scribbler (rabiscador) |
| **BNCSC** | **Broker (corretor)** | Brokerage (corretagem) | Attendant (assistente) |
| **BNCSC** | **Amuse (divertir)** | Amusement (divertimento) | Runner (corredor) |
| **BCSNC** | **Accuse (acusar)** | Accusation (acusação) | Cordially (cordialmente) |
| **BCSNC** | **Doctor (doutor)** | Doctorate (doutorado) | Abolishment (abolição) |
| **BCSNC** | **Rare (raro)** | Rarely (raramente) | Heroic (heróico) |
| **BCSNC** | **Honor (honra)** | Honorable (honorável) | Pollution (poluição) |
| **BCSNC** | **Define (definir)** | Definition (definição) | Decently (decentemente) |
| **BCSNC** | **Salt (salgar)** | Salty (salgado) | Richness (riqueza) |
| **BCSNC** | **Obvious (óbvio)** | Obviously (obviamente) | Virtually (virtualmente) |
| **BCSNC** | **Cruel (cruel)** | Cruelly (cruelmente) | Calmly (calmamente) |
| **BCSNC** | **Dance (dançar)** | Dancer (dançarino) | Isolation (isolamento) |
| **BCSNC** | **Relax (relaxar)** | Relaxation (relaxaçamento) | Firmly (firmemente) |
| **BCSNC** | **Observe (observar)** | Observation (observação) | Punishment (punição) |
| **BCSNC** | **Simple (simples)** | simplicity (simplicidade) | Magician (mágico) |
| **BCSNC** | **Charm (charme)** | Charming (charmoso) | Delicacy (delicadamente) |
| **BCSNC** | **Adapt (adaptar)** | Adaptable (adaptável) | Attacker (atacante) |
| **BNCSNC** | **State (declarar)** | Statement (declaração) | Learner (aprendiz) |
| **BNCSNC** | **Salute (saudar)** | Salutation (saudação) | Stability (estabilidade) |
| **BNCSNC** | **Link (ligar)** | Linkage (ligação) | Header (cabeçalho) |
| **BNCSNC** | **Blind (cego)** | Blindly (cegamente) | Requirement (exigência) |
| **BNCSNC** | **Thumb (polegar)** | Thimble (dedal) | Clientage (freguesía) |
| **BNCSNC** | **Engage (noivar)** | Engagement (noivado) | Politely (educadamente) |
| **BNCSNC** | **Ash (cinza)** | Ashtray (cinzeiro) | Shortage (escassez) |
| **BNCSNC** | **Womanize (mulher)** | Womanizer (mulherengo) | Measurement (medição) |
| **BNCSNC** | **Fever (febre)** | Feverish (febril) | Placement (localização) |
| **BNCSNC** | **Nourish (nutrir)** | Nourishment (nutrição) | Shrinkage (encolhimento) |
| **BNCSNC** | **Milk (leite)** | Milky (leitoso) | Exemption (isenção) |
| **BNCSNC** | **Grade (classificar)** | Grading (classificação) | Concerment (preocupação) |
| **BNCSNC** | **Attain (realizar)** | Attainment (realização) | Marriage (casamento) |
| **BNCSNC** | **Settle (liquidar)** | Settlement (liquidação) | Leakage (vazamento) |
| **pseudoword** | **Werk** | Worker (trabalhador) | Frustration (frustração) |
| **pseudoword** | **Gell** | Teller (contador) | Coverage (cobertura) |
| **pseudoword** | **Dow** | Mower (segador) | Confusable (confundível) |
| **pseudoword** | **Frawl** | Crawler (rasteador) | Evidence (evidência) |
| **pseudoword** | **Alknewledge** | Acknowledgement (reconhecimento) | Famous (famoso) |
| **pseudoword** | **Mign** | Signature (assinatura) | accessible (accesível) |
| **pseudoword** | **Adree** | Agreement (acordo) | Adorable (adorável) |
| **pseudoword** | **Dilgrim** | Pilgrimage (romagem) | Applicable (aplicável) |
| **pseudoword** | **Sommit** | commitment (compromisso) | Collapsible (desmontável) |
| **pseudoword** | **Dakelop** | Development (desenvolvimento) | Communicable (comunicável) |
| **pseudoword** | **Tead** | Leader (líder) | Computer (computador) |
| **pseudoword** | **Sefer** | Deferment (adiamento) | Sinkable (afundável) |
| **pseudoword** | **Leat** | Beater (batedor) | Intruder (intruso) |
| **pseudoword** | **Vurn** | Burner (queimador) | Cleaner (limpador) |
| **pseudoword** | **Ged** | Reddish (avermelhado) | fertilizer (fertilizante) |
| **pseudoword** | **Atrive** | Arrival (chegada) | disposable (descartável) |
| **pseudoword** | **Bood** | Goodness (bondade) | executable (excutável) |
| **pseudoword** | **Ensol** | Enrolment (inscrição) | Explainable (explicável) |
| **pseudoword** | **Avaze** | Amazing (surpreendente) | Digestible (digestível) |
| **pseudoword** | **Boison** | Poisonous (venenoso) | Gradable (graduável) |
| **pseudoword** | **Huppy** | Happiness (felicidade) | Inflatable (insuflável) |
| **pseudoword** | **Obredge** | Abridgement (abreviação) | Lamentable (lamentável) |
| **pseudoword** | **Undertain** | Entertainment (diversão) | Movable (amovível) |
| **pseudoword** | **Joam** | Foamy (espumante) | Permissive (permissivo) |
| **pseudoword** | **Sloom** | Blooming (florescente) | Preciosity (preciosismo) |
| **pseudoword** | **Twamp** | Swampy (pantanoso) | Sugary (açucarado) |
| **pseudoword** | **Caud** | Laudable (loável) | Failed (fracassado) |
| **pseudoword** | **Rud** | Muddy (lamazento) | Bailor (depositante) |
| **pseudoword** | **Pend** | tendency (tendência) | Residence (residência) |
| **pseudoword** | **Iccur** | Occurrence (ocorrência) | resistance (resistência) |
| **pseudoword** | **Kest** | Pestilence (pestilência) | responsible (responsável) |
| **pseudoword** | **Jefer** | Reference (referência) | Armory (armeiro) |
| **pseudoword** | **Talevant** | Relevance (relevância) | Soaker (chuvada) |
| **pseudoword** | **Plex** | Flexible (flexível) | Appearance (aparência) |
| **pseudoword** | **Alevate** | Elevation (elevação) | Importance (importância) |
| **pseudoword** | **Jax** | Taxable (taxável) | Itchy (sarnento) |
| **pseudoword** | **Deutral** | Neutrality (neutralidade) | Eater (comedor) |
| **pseudoword** | **Oclige** | Obligation (obrigação) | Naturally (naturalmente) |
| **pseudoword** | **Uppose** | Opposition (oposição) | Connection (ligação) |
| **pseudoword** | **Tervert** | Perversion (perversão) | Reflection (reflexão) |
| **pseudoword** | **Meduce** | Reduction (redução) | Booster (reforço) |
| **pseudoword** | **Pevise** | Revision (revisão) | Estimation (estimativa) |
| **pseudoword** | **Dound** | Founder (fundador) | Ranker (espesso) |
| **pseudoword** | **Elign** | Alignment (alinhamento) | Blocker (bloqueador) |
| **pseudoword** | **Bove** | Movement (movimento) | Wetting (molhadela) |
| **pseudoword** | **Pirect** | Director (diretor) | Beneficence (beneficiência) |
| **pseudoword** | **Byg** | Baggage (bagagem) | Windy (ventoso) |
| **pseudoword** | **Rycle** | Cyclist (ciclista) | Growler (resmungão) |
| **pseudoword** | **Tave** | Pavement (pavimento) | Branchy (ramoso) |
| **pseudoword** | **Entelop** | Envelopment (envolvimento) | Sweaty (suado) |
| **pseudoword** | **Pation** | National (nacional) | Traveller (viajante) |
| **pseudoword** | **Aject** | Ejector (ejetor) | Illustrate (ilustrar) |
| **pseudoword** | **Droma** | Dramatic (dramático) | Bloody (sangrento) |
| **pseudoword** | **Tenerate** | Generator (generador) | Sleepy (sonolento) |
| **pseudoword** | **Broduce** | Producer (produtor) | Urinate (urinar) |
| **pseudoword** | **Varculate** | Calculator (calculador) | Expected (esperado) |
