# Supplementary figures and images for: The impact of cognateness of word bases and suffixes on morpho-orthographic processing: A masked priming study with intermediate and high-proficiency Portuguese-English bilinguals
Source: PLoS One. 2018 Mar 12;13(3):e0193480. doi: 10.1371/journal.pone.0193480 (PMC5846768; doi:10.1371/journal.pone.0193480)

BCSC

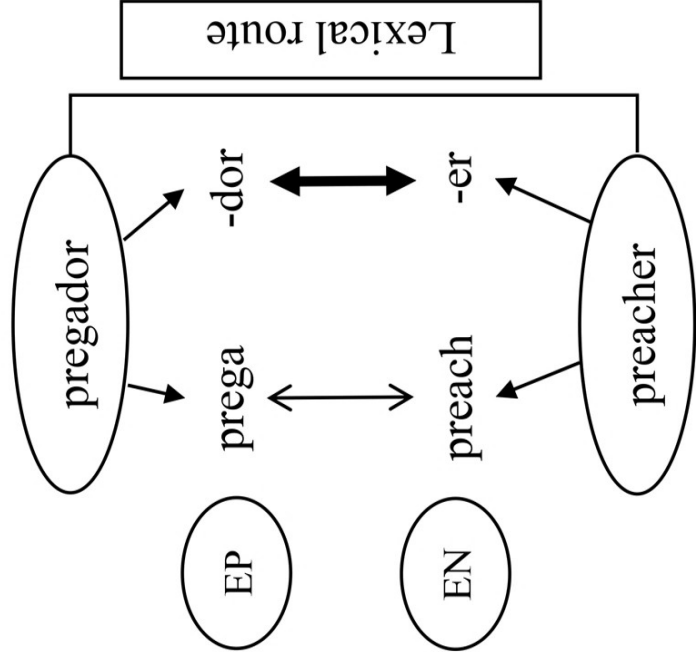

BNCSC

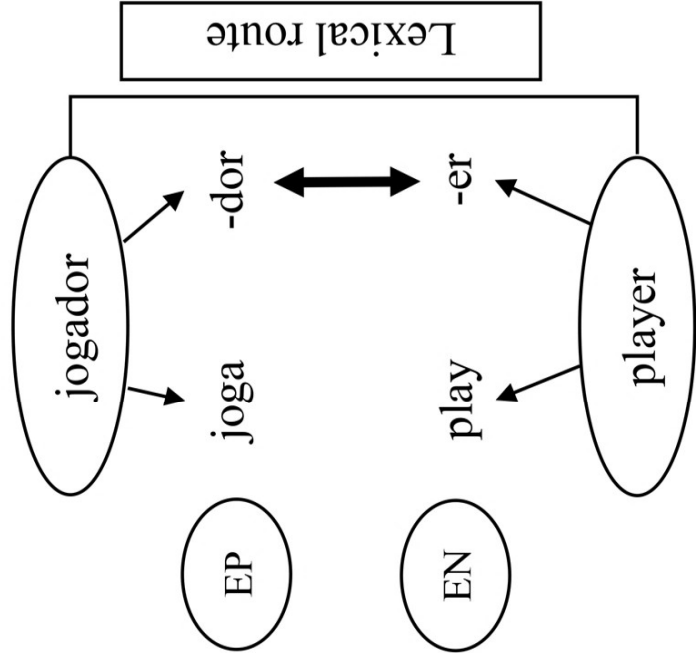

BCSNC

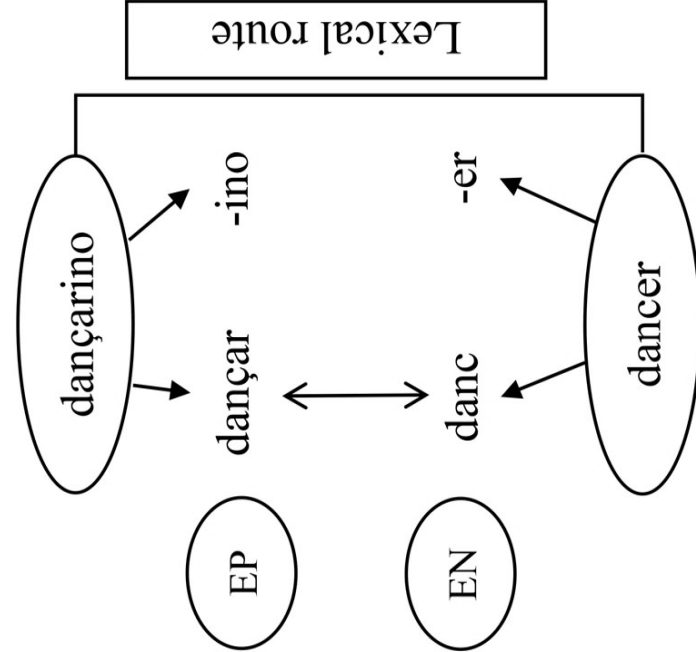

BNC SNC

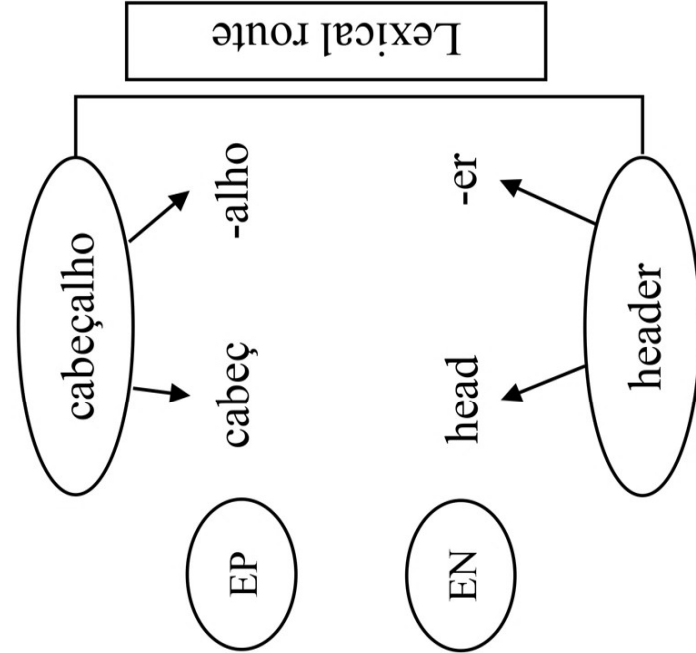

Supplement: S1 Fig — Inputs per word-type are in English (L2): BCSC (preacher, meaning pregador in EP [L1]); BNCSC (player, meaning jogador); BCSNC (dancer, meaning dançarino); and BNCSNC (header, meaning cabeçalho). B stands for Base, S stands for Suffix, C stands for Cognate and NC stands for Non-cognate. Arrows represent facilitatory connections. Stronger links are represented by thick arrows. (PDF) [file pone.0193480.s003.pdf]
